# Altered Resting Functional Connectivity Is Related to Cognitive Outcome in Males With Moderate-Severe Traumatic Brain Injury

**DOI:** 10.3389/fneur.2018.01163

**Published:** 2019-01-10

**Authors:** Nikos Konstantinou, Eva Pettemeridou, Emmanuel A. Stamatakis, Ioannis Seimenis, Fofi Constantinidou

**Affiliations:** ^1^Department of Rehabilitation Sciences, Cyprus University of Technology, Limassol, Cyprus; ^2^Center for Applied Neuroscience, University of Cyprus, Nicosia, Cyprus; ^3^Department of Psychology, University of Cyprus, Nicosia, Cyprus; ^4^Division of Anaesthesia, University of Cambridge, Cambridge, United Kingdom; ^5^Medical Physics Laboratory, Medical School, Democritus University of Thrace, Alexandroupoli, Greece

**Keywords:** traumatic brain injury, resting state, functional connectivity, cognitive outcome, intrinsic connectivity contrast

## Abstract

TBI results in significant cognitive impairments and in altered brain functional connectivity. However, no studies explored so far, the relationship between global functional connectivity and cognitive outcome in chronic moderate-severe TBI. This proof of principle study employed the intrinsic connectivity contrast, an objective voxel-based metric of global functional connectivity, in a small sample of chronic moderate-severe TBI participants and a group of healthy controls matched on gender (males), age, and education. Cognitive tests assessing executive functions, verbal memory, visual memory, attention/organization, and cognitive reserve were administered. Group differences in terms of global functional connectivity maps were assessed and the association between performance on the cognitive measures and global functional connectivity was examined. Next, we investigated the spatial extent of functional connectivity in the brain regions found to be associated with cognitive performance, using traditional seed-based analyses. Global functional connectivity of the TBI group was altered, compared to the controls. Moreover, the strength of global functional connectivity in affected brain areas was associated with cognitive outcome. These findings indicate that impaired global functional connectivity is a significant consequence of TBI suggesting that cognitive impairments following TBI may be partly attributed to altered functional connectivity between brain areas involved in the specific cognitive functions.

## Introduction

Traumatic brain injury (TBI) represents a major medical, public health and socioeconomic problem worldwide (1–3). According to the World Health Organization (WHO), TBI will surpass many diseases as the major cause of death and disability by the year 2020. Indeed, it has been estimated that globally at least 10 million people per year sustain a TBI that is serious enough to result in death or hospitalization. The number of people who have been hospitalized with at least one TBI has been estimated at 57 million, but the proportion of those living with TBI-related disability is still unknown (2).

Approximately 10% of patients with mild TBI, 66% of patients with moderate TBI and all patients with severe TBI will require extensive and costly rehabilitation services due to the effects of their injury (4). In general, TBI is more common in young adults which places a high burden on society because of many life years lost due to disability. TBI results in the greatest number of years lived with a disability due to trauma in Europe (5, 6); the annual cost of traumatic brain injuries is estimated at approximately US$400 billion (7).

Much research has documented that patients with TBI have reduced capacity for activities including functional independence, studying, employment, leisure activities, as well as for personal and social relationships (8–13). Perhaps most importantly, TBI results in significant cognitive dysfunctions, such as attention deficits, memory impairments, and executive functions problems, which relate to mental slowness and reduced processing speed (14–18). Such impairments are thought to be the result of structural brain damage that occurs during the early stage of TBI but importantly, as we now know, continues during the chronic stages of the disease (19, 20).

Recent work indicates that patients with moderate-severe TBI exhibit significant alterations in gray matter volume that are associated with cognitive deficits (21). These effects were found to persist during the chronic stages and at several years post injury supporting the notion that TBI is a long-term condition with chronic implications rather than a static condition following a short recovery phase. Other studies using diffusion tensor imaging (DTI) and/or functional magnetic resonance imaging (fMRI) demonstrated that TBI damages white matter tracts altering structural brain connectivity, which, in turn, may impact functional brain connectivity (22–27).

Evidence of impaired white matter tracts connecting distant brain regions that comprise structural brain networks raises the question as to how TBI affects the functional connectivity of brain areas critical for cognitive functions. Indeed, several recent studies, demonstrated that these abnormalities correlate with the cognitive impairment of patients with mild TBI and are predictive of the cognitive recovery of these patients following a rehabilitation period (23, 28–33).

Despite such plethora of evidence demonstrating altered brain functional connectivity in mild TBI, evidence examining the relationship between cognitive impairment and functional connectivity in patients with moderate-severe chronic TBI is scarce. Previous studies on moderate-severe TBI focused mainly on investigating long-distance interactions between remote brain regions that produce distributed brain networks with distinct functions, termed intrinsic connectivity networks (ICNs). In traditional seed-based analyses, brain areas within ICNs show highly consistent interactions in a pattern that reflects the underlying anatomical structure of white matter connections. A number of ICNs have been identified with abnormalities in TBI patients, including the default mode network [DMN; V. (34–38)], the motor network (39), the thalamic network (40, 41), the executive control network (39, 42), as well as interhemispheric functional connectivity (43).

However, one limitation of this approach is that it restricts analyses due to the requirement to a priori identify ROIs in seed-based analyses. Instead, the intrinsic connectivity contrast [ICC; (44)] has been recently suggested as an approach which allows identification of brain areas with altered functional connectivity without any a priori information that can subsequently be used as seeds in traditional seed-based analyses. Once this is done, the relationship between brain areas with altered functional connectivity and cognitive performance can be examined and contrasted with healthy controls. Our purpose in this proof-of-principle study was to investigate whether this approach can be useful in identifying the relationship between brain areas with altered functional connectivity and their relationship to cognitive outcome in participants with moderate-severe TBI.

We collected resting-state fMRI data and employed the ICC index, a whole-brain voxel-based measure that produces global functional connectivity maps. The ICC index relies on network theory representing the connectivity of each single voxel to the rest of the gray matter voxels in the brain based on the presence of functional connections and the strength of such connections. As mentioned above, in contrast to the traditional ROI methods that require a priori knowledge for the choice and the selection of the ROIs, this index does not require any a priori information or assumptions (44, 45). Our first main aim was to calculate the within-group functional connectivity maps of the participants with TBI and the matched healthy control groups. We computed the ICC relationship with the cognitive measures of executive functions, verbal memory, visual memory, attention/organization, and cognitive reserve, reflecting areas of deficits documented in the chronic TBI literature (15, 21, 46). Following this step, we examined between-group differences in the spatial extent of functional connectivity in the brain regions found to be associated with cognitive performance, using traditional seed-based analyses.

This proof of principle study can contribute toward understanding the neural correlates of cognitive impairments in patients with brain injury. We hypothesized that the global functional connectivity patterns of participants with TBI as indexed by the ICC would be impaired (compared to healthy controls), and that local deficits would include areas associated with the cognitive outcome of the participants with TBI.

## Materials and Methods

### Participants

Eleven participants with TBI were matched to eleven neurologically healthy participants on age, gender and education. All participants were right-handed. Two participants with TBI were subsequently excluded from the analysis due to technical difficulties (one participant was claustrophobic and could not enter the bore of the MRI scanner and the MR images of another participant with TBI were presented with significant motion artifacts rendering the imaging data unusable). The participants with TBI were referred from collaborating physicians using a rolling admission process. Table 1 provides the demographics, time since injury (time lapse between TBI and MRI scan), and injury causes for the remaining nine participants with TBI. Primary causes of TBI were as follows: 44% of the participants (4 participants with TBI) were injured in motor vehicle accidents and another 33% (3 participants with TBI) were injured as a result of work-related falls. The remaining 22% (2 participants with TBI) were injured as a result of falling objects and pedestrian-vehicle collision. All of the TBI participants showed microbleeds on the fluid-attenuated inversion recovery (FLAIR) images. None of the healthy participants showed any microbleeds.

**Table 1:** Demographics and mechanisms of injury for TBI participants.

**TBI**	**Gender**	**Age**	**Education**	**GOSe**	**Days in hospital**	**TSI (months)**	**Antiepileptic drugs**	**Mechanism of injury**
1	M	24	15	4	36	60	Yes	Motor cycle injury
2	M	29	12	8	28	166	No	Fall (work or other non-sports related injury)
3	M	29	14	5	70	176	No	Fall (work or other non-sports related injury)
4	M	60	11	5	7	156	Yes	Motor vehicle crash
5	M	24	14	3	61	84	No	Pedestrian with vehicle collision
6	M	47	12	6	30	274	No	Motor vehicle crash
7	M	30	18	7	30	179	Yes	Motor cycle injury
8	M	35	17	7	25	27	No	Fall (work or other non-sports related injury)
9	M	29	16	8	7	24	Yes	Object fall

*M, male; GOSe, Glaston Outcome Scale Extended; TSI, Time since injury*.

In the present study we recruited only male participants with TBI who had not received any systematic post-acute rehabilitation, which allowed us to avoid the confounding effects of sex and systematic post-injury rehabilitation. Previous research has shown that systematic post-injury rehabilitation results in significant improvements in cognitive functioning of people with TBI (15, 47, 48), whereas research on the effects of sex on cognitive outcome in people with TBI is very limited and often contradictory (49–51).

The inclusion/exclusion criteria included a primary diagnosis of a moderate-to-severe head injury which was determined by at least three of the following indices: (1) initial Glasgow Coma Scale score of <12, (2) abnormal initial computed tomography (CT) or MRI findings indicating acute central nervous system pathology, (3) length of impaired consciousness >20 min as specified by the emergency records, (4) length of post-traumatic amnesia >24 h as specified in the acute hospital/emergency records, (5) length of acute hospital stay >3 days, (5) abnormal neurological examination on hospital admission and discharge indicating focal sensory and motor deficits, or changes in mental status attributed to brain injury, (6) medical complications secondary to the brain injury, and (7) head injury classification as moderate-severe according to hospital records. Other inclusion criteria consisted of the Rancho Los Amigos Scale Level VI or higher (which indicates appropriate, goal-oriented behavior, and post-traumatic amnesia resolution). Additionally, time since injury was at least 24 months prior to the study recruitment (mean = 127.33, *SD* = 83.68 months in the studied group). All participants were native speakers of the Greek language with an age range of 24 to 60 years old (*M* = 34.11, *SD* = 11.92), whilst their education ranged from 11 to 18 years (*M* = 14.12, *SD* = 2.40). None of the participants had received systematic and comprehensive post-acute rehabilitation in the past or at the time of study recruitment. Some of the participants received inpatient rehabilitation services and fragmented individualized outpatient treatment during the acute phase of their recovery. All participants were residing at home at the time of study participation.

The exclusion criteria consisted of the presence of a penetrating head injury, a diagnosis of a stroke at the time of injury, a premorbid central nervous system disorder or learning disability, a premorbid major depression or other significant psychiatric disorder as defined by the Diagnostic and Statistical Manual of Mental Disorders (DSM-V), (52) an active or current alcohol, drug or other controlled substance abuse that would interfere with participation in the study and presence of aphasia with the exception of mild to moderate word finding problems.

All participants in the healthy control (HC) group were Greek-speaking males with no history of a neurological condition or brain trauma, documented psychiatric history, learning disability, or substance abuse. The HC group had an age range of 23 to 60 years old (*M* = 36.55, *SD* = 11.18) and education ranged from 8 to 17 years (*M* = 13.36, *SD* = 2.98).

Each participant gave written informed consent prior to his participation in the study, as approved by the Cyprus National Bioethics committee.

### Neuropsychological Measures

Neuropsychological performance was assessed with a battery of neuropsychological tests which comprised the following conceptually motivated constructs: executive functions, verbal memory, visual memory, attention/organization and cognitive reserve.

The executive functions construct included the Symbol Digits Modalities Test (53), the Trail Making Tests A and B (54) and the Control Oral Word Association Test [Animal naming and words from letter F; (55)].

The Verbal Memory construct included the Greek adaptation of the Auditory Verbal Learning Test [total score in trials 1-5, difference score between trial 5 and trial 1, short delay free recall, long delay free recall, and list A true positive recognition score; (46)] the Digit Span Forward and Backwards total score [adapted Wechsler Memory Scale-Revised, WMS-R (56)], and the Greek adaptation of the paragraphs from the WMS-R Logical Memory I and II [sum of the score of the free recall and the sum of the delayed recall; (56)].

The Visual Memory construct included the Rey Complex Figure Test [immediate recall, delayed recall, recognition total score (57)], the Visual Span Forward and Backwards [from the WMS-R (56)], the spatial visual short-term memory (VSTM) capacity estimate, and the object VSTM capacity estimate [adapted from (21)]. For the spatial and object VSTM capacity tasks, each participant's capacity was assessed using a staircase procedure that estimates the number of spatial locations and the number of objects that a participant can keep in VSTM.

The Attention/Organization construct included the Rey Complex Figure Test [copy and time to copy (57)], and the Distractibility index and the mean reaction time (RT) in a response competition task (21, 58).

The Cognitive Reserve construct included the Pseudowords test [adapted from the Wechsler Individual Achievement Test Second Edition; WIAT-II (59)], and the Peabody Picture Vocabulary Test [Greek adaptation from the PPVT-4; (60, 61)].

### Standard Score Transformation

We followed a standard procedure for calculating composite scores by combining scores from the various tests into the conceptually motived constructs [e.g., (21, 62)], although the validity and reliability of this approach was not tested herein. This approach greatly facilitates examining the association between performance on the cognitive measures and global functional connectivity. Performance scores from each of the relevant cognitive and experimental tests are combined into composite scores representing the conceptually motivated constructs of executive functions, verbal memory, visual memory, attention/organization, and cognitive reserve. Each participant's score from each of the individual measures was transformed into a standard score (z-score) based on the mean and the standard deviation of the HC group, in order to allow group comparisons. For this reason, the mean score of the HC group in Table 2 is omitted.

**Table 2:** Performance of TBI participants on cognitive measures.

**Measure**	**Mean (*SD*)**	***t***	***df***	***P***	**Cohen's *d***
Verbal Memory	−0.77 (0.93)	2.12	18	0.025	0.87
Visual Memory	−0.73 (1.36)	1.63	18	0.060	0.70
Executive Functions	−3.10 (4.47)	2.29	18	0.017	0.93
Attention/Organization	−1.46 (1.79)	2.50	18	0.011	0.99
Cognitive Reserve	−0.90 (1.69)	1.56	18	0.070	0.67

*SD, standard deviation; df, degrees of freedom; P, one-tailed*.

Standard scores from tasks where a higher score indicated worst performance (e.g., response times) were transformed by being multiplied with minus one such that higher scores in all tasks indicated better performance. The resulting standard scores from each of the measures were then averaged together to derive a score for each of the constructed measures.

### MRI Data Acquisition and Analysis

MR images were acquired with a 3.0-T scanner (Achieva, Philips Medical Systems, Best, The Netherlands). The built-in quadrature RF body coil and a phased array 8-channel head coil were used for proton excitation and signal detection, respectively. The scanning session included other standard pulse sequences [e.g., T1-weighted rapid acquisition gradient-echo, T2-weighted turbo spin echo, diffusion weighted imaging, diffusion tensor imaging, fluid-attenuated inversion recovery (FLAIR) and susceptibility-weighted imaging (SWI)] to exclude significant brain pathology of a different etiology.

fMRI assessment involved a resting state scan with series of 160 volumes for which participants were instructed to not think of anything in particular and to keep their eyes open. After the scanning session, participants confirmed they had kept their eyes open during the scan and had not fallen asleep. None of the participants underwent an MRI under general anesthesia or sedation. Data were acquired using a spin echo EPI (echo-planar imaging) pulse sequence with the following parameters: TR = 3,000 ms, TE = 70 ms, flip angle = 90°, acquisition voxel size = 2.4 × 2.4 × 4.0 mm, reconstruction voxel size = 1.8 × 1.8 × 4.0 mm, slices per volume = 28.

Prior to preprocessing, the first five fMRI volumes were removed to eliminate saturation effects and achieve steady-state magnetization. Images were preprocessed using Statistical Parametric Mapping 12 (SPM12) (Wellcome Trust Center for Neuroimaging, http://www.fil.ion.ucl.ac.uk/spm/software/spm8/) implemented in MatLab R2015 (Mathworks, Natick, MA, USA). Images were slice time corrected, realigned and unwarped, coregistered (without reslice) to the individual participant's morphological scan, spatially smoothed with a narrow Gaussian kernel of 8 mm at full width half maximum FWHM, and spatially normalized to a standard EPI template in the Montreal Neurological Institute (MNI) space. Visual inspection after every step was performed to ensure appropriate quality of preprocessing. The CONN fMRI functional connectivity toolbox (63) was used to calculate the intrinsic connectivity contrast (ICC) of each participant. Before the ICC calculation we used CompCor, a strict noise reduction method, to remove data components attributable to the signal from white matter and cerebrospinal fluid (64). The method is based on white matter and cerebrospinal fluid masks from the T1-weighted segmented images and eliminates the need for global signal normalization (65, 66). The demographic factors of Age and Education, and the six subject-specific realignment parameters with their first order derivatives were also factored-out before calculating the ICC index (67). A temporal filter of 0.009 and 0.08 Hz was applied to focus on low-frequency fluctuations (68).

Anatomical brain regions were found using SPM Anatomy toolbox (69, 70) and MRIcroN (71).

### Intrinsic Connectivity Contrast Analyses

The Intrinsic Connectivity Contrast (ICC) is a voxel-wise index (a single number for each voxel) that represents how well-connected each voxel is to the rest of the gray matter in the brain (44). Following the calculation of the resting state ICC map for each participant, the images were entered into a single multiple regression analysis in SPM12. The design matrix of the model included the following regressors: group (TBI vs. HC) and one regressor for each of the cognitive measures (i.e., the composite scores of executive function, verbal memory, visual memory, attention/organization and cognitive reserve). The design matrix also included age, years of education, and time since injury as regressors of no interest. To capture any motion-related artifacts, motion parameters were also included in the model. Sex was not included in the design matrix since all participants were males. Automatic orthogonalization in SPM was applied to address the problem of collinear regressors in the model. Following false discovery rate (FDR) correction for multiple comparisons across the whole brain, a statistical threshold of *p* < 0.05 was used.

Associations between the estimated neuropsychological measures and ICCs were tested using a voxel–wise approach within the general linear model framework. Specifically, we assessed the whole brain correlation between each neuropsychological measure and the voxel level ICC of each group, as well as the interaction between the neuropsychological measure and the variable Group (participants with TBI vs. HC) in the multiple regression model described above.

### Seed-Based Analyses

The ICC index is a global functional connectivity index and does not provide information on the spatial extent of functional connectivity. For this reason and, in order to calculate within-group functional connectivity maps of the regions found to be associated with each of the neuropsychological measures, we employed seed-based analysis using the CONN fMRI toolbox. This analysis also allowed us to examine between-group (participants with TBI vs. HC) spatial differences in network integrity.

## Results

### Demographics and MRI

Two-tailed, two-sample *t*-tests revealed that the groups with TBI and HC participants were very similar in terms of age and education (all *t* < *1*). Any significant differences in the following comparisons between the two groups cannot, thus, be attributed to sample differences in terms of gender, age or education.

Structural and morphological imaging findings in TBI participants included atrophy, impaired white mater integrity, gliosis, and haemosiderin depositions (demonstrated in SWI images). All of the above findings are compatible with chronic head trauma. None of the healthy participants showed any microbleeds. Neither superficial siderosis nor other significant pathology was detected in any participant.

### Cognitive Measures

One-tailed independent samples *t-*tests were conducted in order to compare the performance of the two groups on the constructed measures of Verbal Memory, Visual Memory, Executive Functions, Attention/Organization, and Cognitive Reserve. As shown in Table 2, compared to the non-injured HC participants, the performance of participants with TBI was significantly lower on the neuropsychological constructed measures of Verbal Memory, Attention, and Executive Functions. The performance on the remaining two measures of Visual Memory (*p* = 0.06) and Cognitive Reserve (*p* = 0.07) did not differ significantly between the two groups.

### Intrinsic Connectivity Contrast

**Figure 1:**
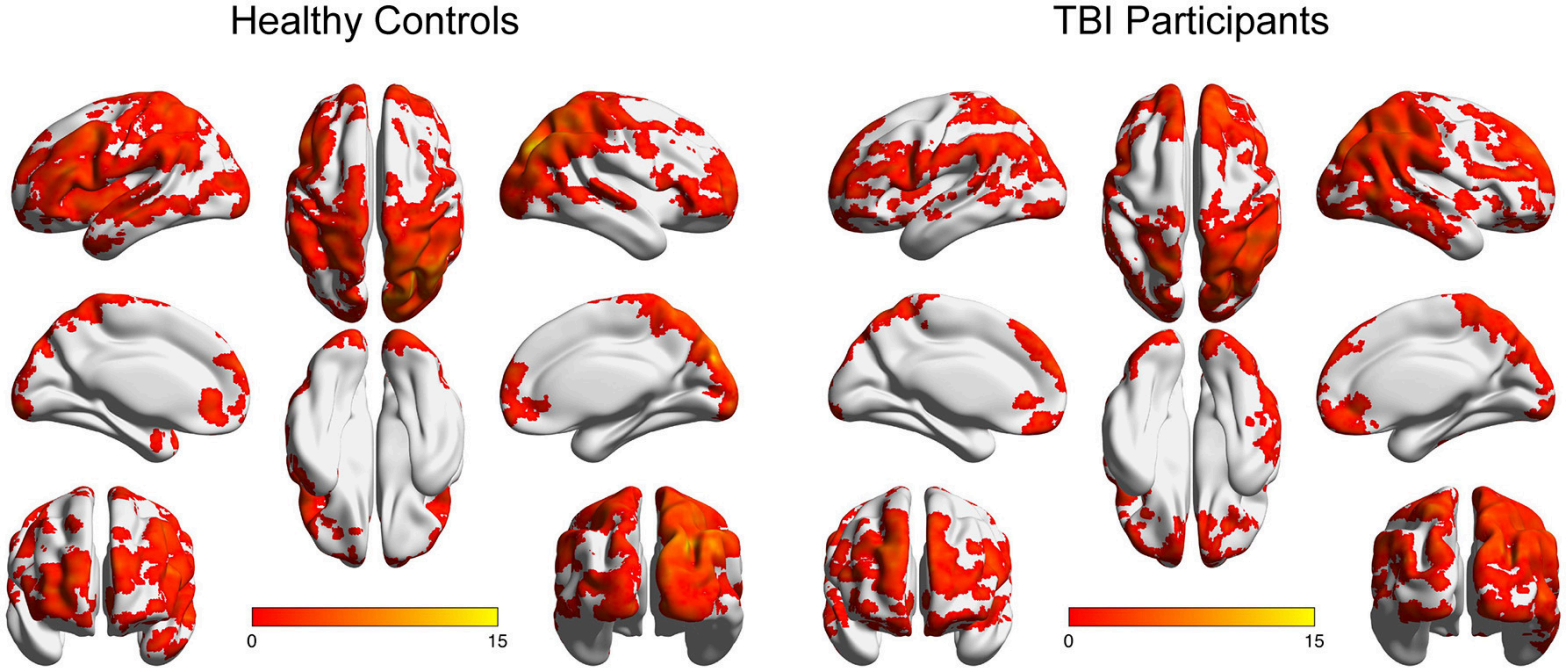
Within-group ICC maps for both groups. Results are presented on inflated brains created using the BrainNet tool (72). Images are displayed in neurological convention (left is left). Color scale represents t-score values.

Figure 1 depicts the within-group ICC maps for the TBI and the HC groups showing that HC brains appear to be more connected than TBI patients' brains. Specifically, in both groups, within-group high ICC was observed in posterior regions including the lateral occipital cortex, the angular gyrus, the occipital pole, the supramarginal gyrus, the superior parietal lobule, and the precuneus. High ICC maps were also observed in the frontal pole, the middle frontal gyrus, and the superior frontal gyrus.

However, of main interest here are specific areas that correlate with the cognitive measures. Several brain areas in the group with TBI, described in Table 3, exhibited significant correlations between the cognitive constructed measures and the corresponding within-group ICC maps. Specifically, the within-group ICC maps at the left medial Temporal pole, exhibited a significant positive correlation with Executive Functions scores, whereas the left Putamen exhibited a significant negative relationship. The within-group ICC maps at the left medial temporal pole, left superior parietal lobule, right inferior frontal gyrus, and the superior part of the right medial frontal gyrus exhibited a significant positive correlation with the Verbal Memory scores, whereas the right precuneus and right angular gyrus exhibited a significant negative relationship with the Verbal Memory scores. The left middle temporal gyrus exhibited a significant positive correlation with Visual Memory scores, whereas the left putamen, left superior orbital gyrus, left hippocampus, left pallidum, and right anterior cingulate cortex (ACC), exhibited a significant negative correlation with Visual Memory scores. The left superior temporal gyrus exhibited a significant positive correlation with Attention scores, whereas the left putamen and right ACC exhibited a significant negative correlation with Attention scores. The left superior temporal gyrus exhibited a significant positive correlation with Cognitive Reserve scores.

**Table 3:** Within-group correlations between ICC and cognitive measures (p-FDR < 0.05 corrected) for the TBI patients.

**Neurocognitive measure**	**Brain region**	**Relationship direction**	**MNI coordinates**			**Cluster size (mm^**3**^)**	**T-value**	**Cluster p-unc**.	**Cluster p-FDR**
			**X**	**Y**	**Z**				
Executive Functions	L Inferior Temporal gyrus	Positive	−40	8	−40	38	9.58	0.000	0.011
	L Putamen	Negative	−10	8	−6	219	−13.30	0.000	0.000
Verbal Memory	L Medial Temporal pole	Positive	−50	8	−28	74	12.36	0.000	0.000
	L Middle Temporal gyrus	Positive	−62	−14	−12	50	10.83	0.000	0.001
	R Inferior Frontal gyrus	Positive	42	30	−22	43	10.70	0.000	0.002
	L Superior Parietal lobule	Positive	−24	−68	64	22	9.28	0.002	0.033
	R Superior Medial Frontal gyrus	Positive	16	62	14	22	6.52	0.002	0.033
	R Precuneus	Negative	30	−56	20	68	−14.18	0.000	0.000
	R Angular gyrus	Negative	42	−46	22	52	−9.54	0.000	0.002
Visual Memory	L Middle Temporal gyrus	Positive	−52	−6	−24	37	7.39	0.000	0.021
	L Putamen	Negative	−18	20	−6	79	−17.08	0.000	0.000
	R Anterior Cingulate	Negative	10	28	−2	77	−11.73	0.000	0.000
	L Superior Orbital Frontal gyrus	Negative	−12	22	−18	66	−10.92	0.000	0.000
	L Hippocampus	Negative	−30	−32	−6	25	−10.47	0.000	0.021
	L Pallidum	Negative	−8	2	−4	21	−8.95	0.002	0.037
Attention	L Superior Temporal gyrus	Positive	−46	10	−24	77	8.48	0.000	0.000
	L Putamen	Negative	−22	14	−6	52	−11.38	0.000	0.002
	R Caudate Nucleus	Negative	12	16	−10	34	−8.44	0.000	0.013
Cognitive Reserve	L Superior Temporal gyrus	Positive	−46	10	−26	61	7.82	0.000	0.000

*L, left hemisphere; R, right hemisphere; p-unc, p-value for the cluster uncorrected; p-FDR, p-value for the cluster corrected for multiple comparisons; T-value, t-value for peak voxel*.

A positive correlation between the ICC score and the score on a composite measure in the above analyses indicates that global connectivity in a specific brain area and the corresponding cognitive function move in the same direction, that is, increased global connectivity of that specific brain area is associated with better ability to exercise the corresponding cognitive function or reduced connectivity is associated with impaired cognitive ability. On the contrary, a negative correlation indicates that global connectivity at a specific brain area and the corresponding cognitive function move in opposite directions, where either increased connectivity is associated with better cognitive ability or reduced connectivity is associated with impaired cognitive ability.

**Figure 2:**
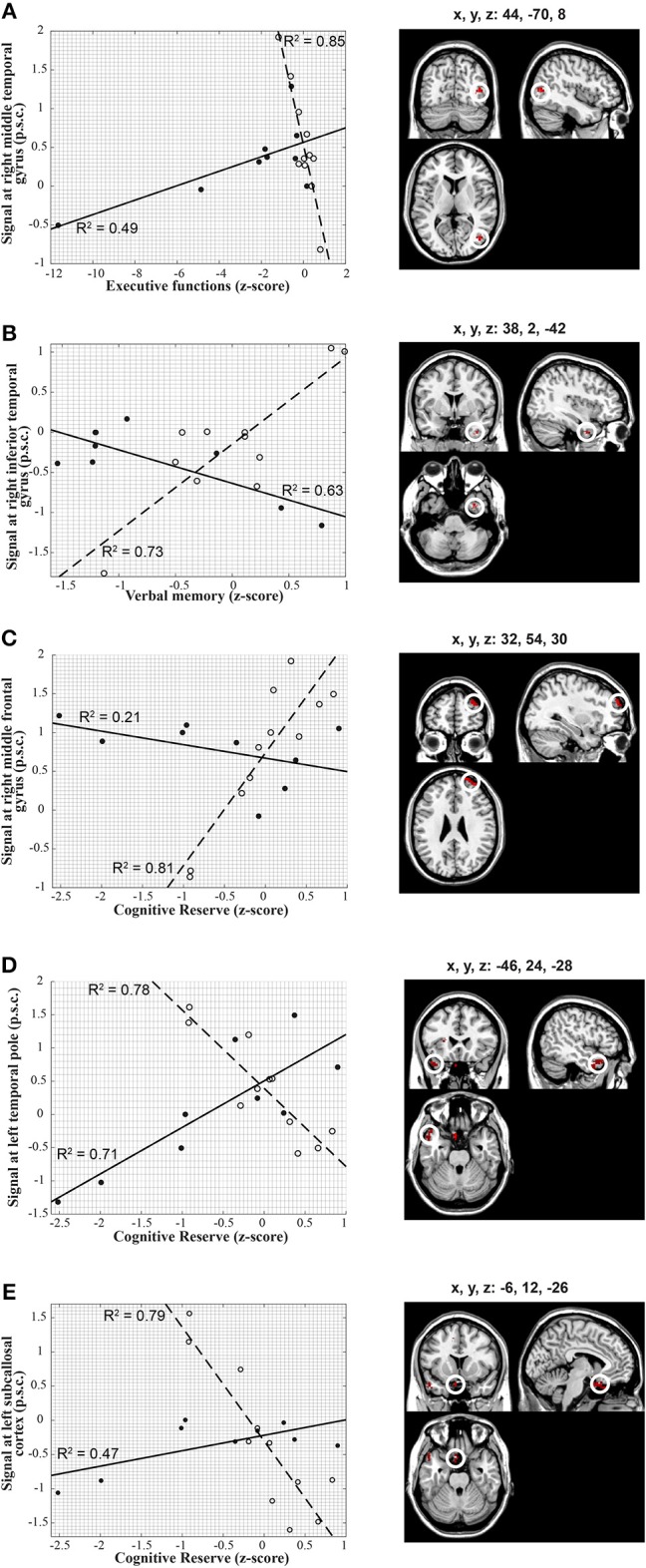
Voxel-wise correlations between ICC extracted from brain regions depicted on the right-hand panels and individual scores in the cognitive constructed measures along with the corresponding scatter plots (left-hand panels). The scatter plots on the left show in detail the relationship between ICC and cognitive measures in each of the statistical peaks for both groups (TBI: solid lines, closed circles, HC: dashed lines, open circles). Results are overlaid onto a standard single subject T1-weighted MR-image (ch2-template) in the MRICroN software (71). **(A)** Correlation between the cognitive measure of executive function and right middle Temporal gyrus in both groups. The correlation of ICC with executive function scores was positive in participants with TBI [*r*_(6)_ = 0.70; *p* = 0.04] and negative in the healthy controls [*r*_(9)_ = −0.92; *p* < 0.001] for the right middle temporal gyrus [peak at x, y, z = 44, −70, 8; *t*_(1, 16)_ = 6.66; p-FDR < 0.05]. **(B)** Correlation between verbal memory and right inferior Temporal gyrus in both groups. The correlation of ICC with verbal memory scores was negative in participants with TBI [*r*_(6)_ = 0.79; *p* = 0.01] and positive in the healthy controls [*r*_(9)_ = 0.85; *p* = 0.001] for the right inferior Temporal gyrus [peak at x, y, z = 38, 2, −42; *t*_(1, 16)_ = 6.01; p-FDR = 0.04]. **(C)** Correlation between cognitive reserve and right middle Frontal gyrus in both groups. The correlation of ICC with cognitive reserve scores exhibited a negative trend in participants with TBI but did not reach statistical significance [*r*_(6)_ = −0.46; *p* = 0.21], whereas it was positive in the healthy controls [*r*_(9)_ = 0.90; *p* < 0.001] for the right middle frontal gyrus [peak at x, y, z = 32, 54, 30; *t*_(1, 16)_ = 6.13; p-FDR < 0.001]. **(D)** Correlation between cognitive reserve and left Temporal pole in both groups. The correlation of ICC with cognitive reserve scores was positive in participants with TBI [*r*_(6)_ = 0.84; *p* = 0.005] and negative in the healthy controls [*r*_(9)_ = −0.88; *p* < 0.001) for the left temporal pole [peak at x, y, z = −46, 24, −28; *t*_(1, 16)_ = 6.46; p-FDR < 0.001). **(E)** Correlation between cognitive reserve and left Subcallosal cortex in both groups. The correlation of ICC with cognitive reserve scores was positive in participants with TBI [*r*_(6)_ = 0.68; *p* < 0.05] and negative in the healthy controls [*r*_(9)_ = −0.89; *p* < 0.001] for the left subcallosal cortex [peak at x, y, z = −6, 12, −26; *t*_(1, 16)_ = 6.80; p-FDR < 0.001]. Percent signal change, p.s.c.

Significant between-group interactions of ICC correlations with cognitive measures are shown in Figure 2. All significant correlations were strong as indicated by all r>0.68. Specifically, the correlation between ICC in the right middle temporal gyrus and Executive Functions was negative in the healthy controls and positive in the participants with TBI (Figure 2A); between ICC in the right inferior temporal gyrus and Verbal Memory was positive in the healthy controls and negative in the participants with TBI (Figure 2B); between ICC in the right middle frontal gyrus and Cognitive Reserve was positive in the healthy controls and negative in the participants with TBI (Figure 2C); between ICC in the left temporal pole and Cognitive Reserve was negative in the healthy controls and positive in the participants with TBI (Figure 2D); between ICC in the left subcallosal cortex and Cognitive Reserve was negative in the healthy controls and positive in the participants with TBI (Figure 2E).

### Seed-Based Analyses

**Figure 3:**
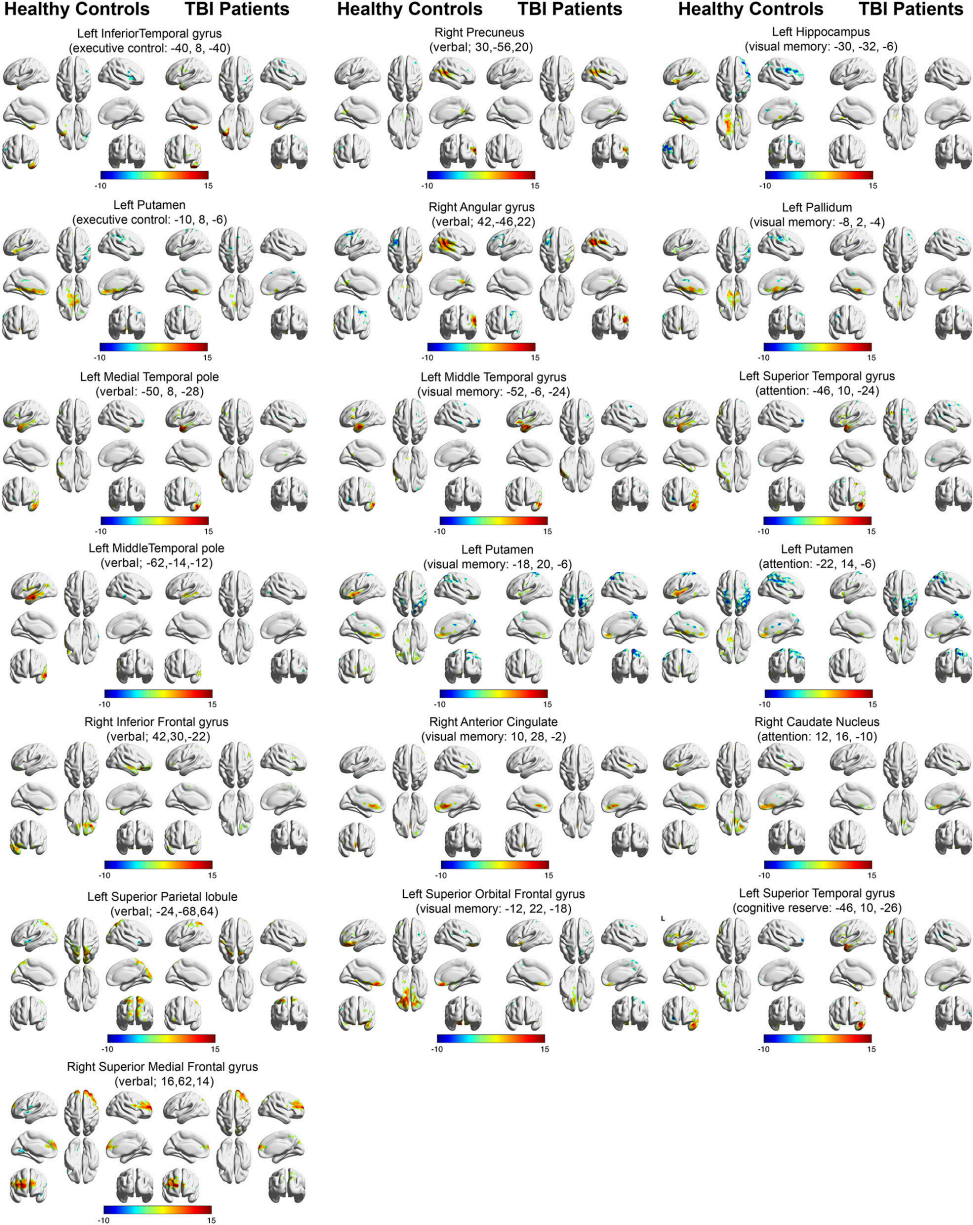
Within-group functional connectivity maps calculated from seed areas showing significant correlations with the TBI participants. Voxels showing significant positive functional connectivity are shown in red–yellow color scale, and voxels showing significant negative functional connectivity are shown in green-blue color scale.

**Figure 4:**
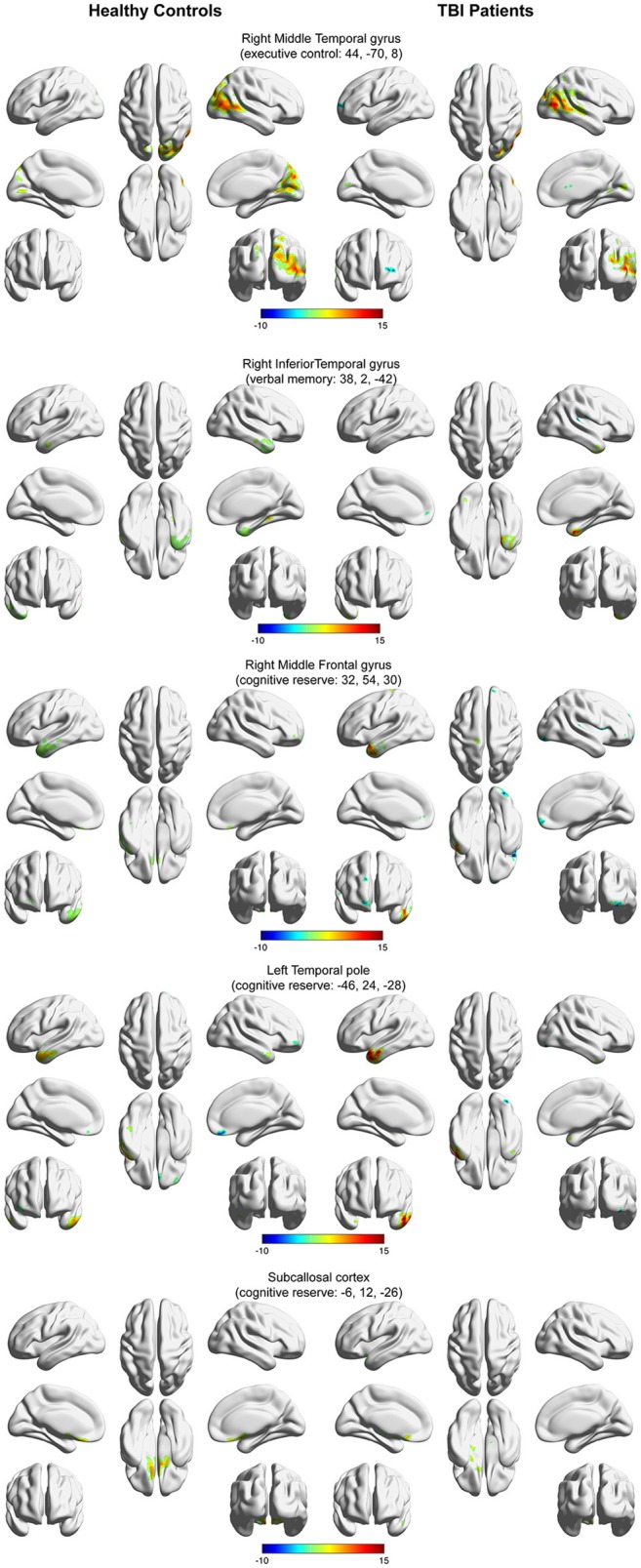
Within-group functional connectivity maps calculated from seed areas showing a significant interaction between the healthy controls and the TBI participants. Voxels showing significant positive functional connectivity are shown in red–yellow color scale, and voxels showing significant negative functional connectivity are shown in green-blue color scale.

Figure 3 depicts the within-group functional connectivity maps of each group calculated using as seeds the regions that exhibited a significant relationship between the TBI within-group ICC maps and each of the neuropsychological measures (see Table 3). Figure 4 depicts the within-group ICC maps calculated from seed areas that displayed a significant between-group interaction with the cognitive measures.

**Figure 5:**
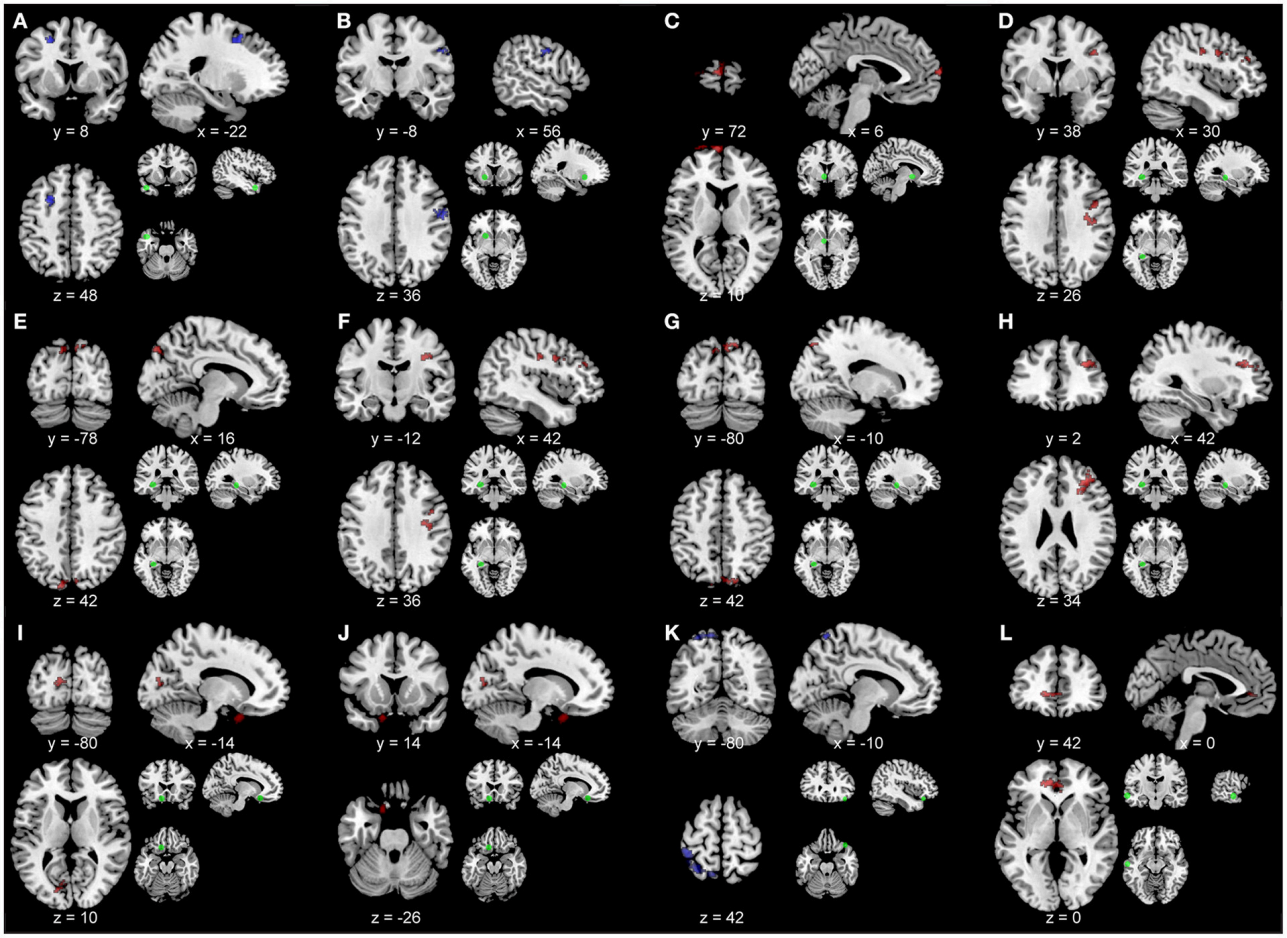
Results of the between-group comparisons of the seed-based analyses overlaid on a template brain. Blue represents greater functional connectivity in the TBI participants compared to the healthy controls. Red color represents greater functional connectivity in the healthy controls compared to the TBI participants. Small brain images with green circles represent the seed area. Results are overlaid onto a standard single subject T1-weighted MR-image (ch2bet-template) in the MRICroN software (71). Images are displayed in neurological convention (left is left). **(A)** Seed, left middle temporal pole. Peak, left middle frontal sulcus at x, y, z = −22, 8, 48; *t*_(1, 16)_ = 3.69; pDFR = 0.022. **(B)** Seed, left putamen. Peak, right postcentral gyrus at x, y, z = 56, −8, 36; *t*_(1, 18)_ = 3.61; pDFR = 0.036. **(C)** Seed, left pallidum. Peak, left superior medial frontal gyrus at x, y, z = 6, 72, 10; *t*_(1, 18)_ = 3.61; pDFR = 0.001. **(D)** Seed, left hippocampus. Peak, right middle frontal sulcus at x, y, z = 30, 38, 26; *t*_(1, 18)_ = 3.61; pDFR = 0.003. **(E)** Seed, left hippocampus. Peak, right precuneus at x, y, z = 16, −78, 48; *t*_(1, 18)_ = 3.61; pDFR = 0.039. **(F)** Seed, left hippocampus. Peak, right precentral sulcus at x, y, z = 42, −12, 36; *t*_(1, 18)_ = 3.61; pDFR = 0.039. **(G)** Seed, left hippocampus. Peak, left superior occipital lobule [peak at x, y, z = −10, −80, 42; *t*_(1, 18)_ = 3.61; pDFR = 0.047]. **(H)** Seed, left hippocampus. Peak, right precentral sulcus at x, y, z = 42, 2, 34; *t*_(1, 18)_ = 3.61; pDFR = 0.047. **(I)** Seed, left superior orbito-frontal gyrus. Peak, left calcarine sulcus [peak at x, y, z = −14, −80, 10; *t*_(1, 18)_ = 3.61; pDFR = 0.048]. **(J)** Seed, left superior orbito-frontal gyrus. Peak, left inferior orbito-frontal gyrus at x, y, z = −14, 14, −26; *t*_(1, 18)_ = 3.61; pDFR = 0.048. **(K)** Seed, right angular gyrus. Peak, left precuneus at x, y, z = −10, −80, 42; *t*_(1, 17)_ = 3.65; pDFR = 0.001. **(L)** Seed, left middle temporal gyrus. Peak, left anterior cingulum at x, y, z = 0, 42, 0; *t*_(1, 18)_ = 3.61; pDFR = 0.017.

Figure 5 shows the between-group functional connectivity differences in the TBI and healthy control groups using as seeds the brain areas with significant relationships between the TBI within-group ICC maps and each of the neuropsychological measures. This analysis revealed that compared to the healthy control group, participants with TBI showed greater functional connectivity between the left middle temporal pole seed and a region in left middle frontal sulcus [peak at x, y, z = −22, 8, 48; *t*_(1, 16)_ = 3.69; pDFR = 0.022; Figure 5A], the left putamen seed and a region in right postcentral gyrus [peak at x, y, z = 56, −8, 36; *t*_(1, 18)_ = 3.61; pDFR = 0.036; Figure 5B], and the right angular gyrus seed and the left precuneus [peak at x, y, z = −10, −80, 42; *t*_(1, 17)_ = 3.65; pDFR = 0.001; Figure 5K]. Moreover, participants with TBI showed reduced functional connectivity (compared to the healthy control group) between the left pallidum seed and a region in left superior medial frontal gyrus [peak at x, y, z = 6, 72, 10; *t*_(1, 18)_ = 3.61; pDFR = 0.001; Figure 5C], the left hippocampus seed and the right middle frontal sulcus [peak at x, y, z = 30, 38, 26; *t*_(1, 18)_ = 3.61; pDFR = 0.003; Figure 5D], the right precuneus [peak at x, y, z = 16, −78, 48; *t*_(1, 18)_ = 3.61; pDFR = 0.039; Figure 5E], the right precentral sulcus [peak at x, y, z = 42, −12, 36; *t*_(1, 18)_ = 3.61; pDFR = 0.039; Figure 5F], the left superior occipital lobule [peak at x, y, z = −10, −80, 42; *t*_(1, 18)_ = 3.61; pDFR = 0.047; Figure 5G], the right precentral sulcus [peak at x, y, z = 42, 2, 34; *t*_(1, 18)_ = 3.61; pDFR = 0.047; Figure 5H], the left superior orbito-frontal gyrus seed with the left calcarine sulcus [peak at x, y, z = −14, −80, 10; *t*_(1, 18)_ = 3.61; pDFR = 0.048; Figure 5I] and the left inferior orbito-frontal gyrus [peak at x, y, z = −14, 14, −26; *t*_(1, 18)_ = 3.61; pDFR = 0.048; Figure 5J], and the left middle temporal gyrus seed and the left anterior cingulum [peak at x, y, z = 0, 42, 0; *t*_(1, 18)_ = 3.61; pDFR = 0.017; Figure 5L].

## Discussion

The main aim of this proof of principle study was to utilize the ICC index to investigate whether changes in functional brain connectivity patterns in moderate-severe TBI are related to cognitive outcome. Although TBI has been previously associated with altered functional brain connectivity and with impaired cognitive functioning, the relationship between these two variables has not been explored. Due to the high prevalence of TBI among males, we focused on a homogeneous group of male participants with moderate-severe chronic TBI that had not received any post-acute systematic comprehensive rehabilitation. Studying this homogeneous group allowed us to gain a more precise understanding of the true relationship between functional connectivity and cognitive outcome in males by avoiding the confounding effects of sex and rehabilitation (15, 47–51). We utilized a novel index (ICC) to determine the global functional connectivity at the level of individual voxels, (73) followed by seed-based analyses to characterize the spatial extend of functional connectivity changes. Our main finding was that the brain areas exhibiting altered integrity of functional brain connectivity at rest in participants with TBI, compared to matched healthy controls, were associated with outcomes in cognitive measures of executive function, verbal memory, visual memory, attention/organization and cognitive reserve.

Within-group maps of the ICC were obtained in posterior cortical areas (e.g., lateral and medial occipital areas), parietal areas (e.g., angular gyrus and superior parietal lobe), frontal lobe areas (including lateral, superior and medial regions), and lateral and inferior temporal lobe areas, for both groups. However, close visual inspection of the within-group functional connectivity maps of participants with TBI and the corresponding maps of healthy controls revealed that participants with TBI exhibited similar maps albeit with more limited spatial extend. This finding demonstrates that the strength of global connectivity between several brain areas and the rest of the brain is altered in chronic moderate-severe TBI, indicating that the impaired global connectivity is a significant consequence of brain injury.

Similar reduced resting-state functional connectivity maps have also been found in patients with mild cognitive impairment and Alzheimer's disease (74). In these neurodegenerative diseases, reduced functional connectivity at rest reflects loss of neurons that consequently affects connectivity and results in breakdown of brain networks. It is suggested, therefore, that the reduced structural brain connectivity [e.g., (75)] and reduced brain volume due to the brain injury [e.g. (21)] that have been observed previously may be responsible for the results of altered global functional connectivity observed in this study. Taken together with earlier findings on the impaired cognitive functioning of the participants with TBI (compared to the healthy controls), this study utilizing the ICC index provides a new line of evidence for the suggestion that the injured brain remains affected for many years after the initial insult.

The findings of the present study, using a similar methodology to a recent report by Moreno-López et al. (73) that characterized the association between depressive symptomatology and global functional connectivity in TBI, complement previous reports in highlighting that altered functional connectivity in chronic TBI may be predictive of cognitive outcome (34–42, 76). Current findings indicate for the first time that the strength of global functional connectivity is related to the cognitive outcome of people with moderate-severe TBI, demonstrating that the ICC index could potentially prove to be a useful imaging biomarker in characterizing and monitoring the TBI impact on cognitive performance.

An additional contribution of the current study may lay in the fact that through combining the ICC index analysis with more traditional seed-based analyses, our findings contribute in the understanding of the relationship between functional connectivity and cognitive processing in chronic moderate-severe TBI, confirming well-established links between executive functioning, verbal memory, visual memory, and cognitive reserve, with frontal, temporal, parietal, cerebellar, and subcortical areas.

Specifically, the regions associated here with performance on executive functions, including the putamen, the left medial temporal pole and the right middle temporal gyrus, have been previously implicated in executive functions, including goal-directed behavior (77), executive control during performance in visuospatial tasks (78), cognitive control during goal-directed mental simulation (79), performance in a planning task (80), and in a divided attention task (81), interference control (82), mental rotation (83), mental updating (84), and task switching and resolution of competition between potentially relevant tasks (85).

With regards to verbal memory, the finding that performance in verbal memory is associated with functional connectivity in temporal lobe regions is consistent with models in which medial temporal cortex is involved in semantic processing across a range of input modalities [including verbal, visual, and tactile; (86–88)]. Moreover, the right inferior frontal gyrus was previously found to be active during reading, semantic, and phonological decision tasks (89) and during deep processing of verbal information in memory (90). The left superior parietal lobule showed significant activity during memory retrieval of verbal information (91) and increased fMRI activity in a letter delayed recognition task (92). These findings support the view that the short-term retention of verbal information is supported by regions that carry out relatively early stages of acoustic, lexical, phonological, and speech-based processing indicating that these brain regions may have a dual function, the short-term maintenance in addition to the precise encoding of this information (93).

Performance in the visual memory, attention/organization and cognitive reserve was related to global functional connectivity in brain areas that were previously shown to exhibit activity during corresponding experimental tasks, confirming well-established links between cognitive performance and brain function. For instance, the left medial temporal gyrus has been involved in delay-period activity in a delayed match to sample short-term memory task for visual information (94), and activity in this area during a similar visual short-term memory task was found to be specifically related only to memory probes indicating that it is specifically involved in visual memory recognition (95). Brain regions with functional connectivity related to attention/organization were found to exhibit activity during attention tasks. For example, in a selective attention task of car experts, the left superior temporal gyrus showed fMRI activity that was specific to the attended condition (96). The left Putamen showed significantly enhanced activity associated with cued attention shifts during an auditory selective attention task (97), and activity in this brain area was specific to covert attention shifts, specifically when participants performed a covert visual assessment of a peripheral stimulus in the absence of any saccades (98). The right Caudate nucleus showed fMRI activity related to the attended condition in a selective attention task of car experts (96). Taken together, these findings demonstrate that impaired global functional connectivity is associated with cognitive outcome and it constitutes an important consequence of brain injury.

Comparing functional connectivity between the two groups revealed that the direction of relationship between global functional connectivity (as measured with the ICC index) and measures of executive functions, verbal memory, and cognitive reserve was found to depend on whether the participants were TBI survivors or healthy controls. These findings indicate that perhaps the reduced structural brain connectivity and reduced brain volume due to the brain injury (19) result in reduced global functional connectivity in some brain areas but also could produce increased global functional connectivity in other brain areas, presumably in a way that compensates for the loss of brain volume and structural brain connectivity. Furthermore, reductions in cognitive performance in chronic moderate-severe TBI have been associated with reductions in white matter and gray matter volume that was not widespread, but followed a fronto-thalamic pattern (21).

The small sample size of the TBI group constitutes the main limitation of the current study. Nevertheless, the validity of the present results is enhanced by the strict inclusion/exclusion criteria adopted, and by the homogeneity of the participants included. Specifically, most previous studies investigating the effects of TBI on brain structure and function are affected by the confounding factors of comprehensive post-injury rehabilitation and sex. Comprehensive post-injury rehabilitation has been shown to improve cognitive functioning in TBI (15, 47, 48). For example, Till et al. (99) showed that the best predictor of cognitive outcome at 2-5 years post-injury was the amount of hours of rehabilitation at 5 months postinjury, independent of injury severity or the initial severity of cognitive impairment. The present study avoided the confounding effects of rehabilitation by recruiting a group of participants that had not received comprehensive post-injury rehabilitation, thus gaining a more accurate understanding of the true effects of chronic TBI on brain connectivity.

Sex constitutes another confounding factor of previous research investigating the chronic effects of TBI on the brain. For example, in animal models of TBI, females demonstrate better outcomes compared to males, supporting the idea that in TBI gonadal steroids (i.e., estrogen and progesterone) may induce neuroprotective effects in TBI (100–103). However, the findings on the effects of sex on cognitive outcome due to TBI in humans are still very limited and with contradictory findings (49, 50, 103, 104). In order to circumvent possible confounding effects of sex and due to the higher prevalence of TBI among males, compared to females (105), the current study focused on a homogeneous group of male TBI participants.

Future work should focus on dissociating the effects of sex and better understand the effects of rehabilitation on brain connectivity. Moreover, future work should investigate the temporal pattern of the functional connectome changes related to moderate-severe TBI and should investigate if the ICC index can be used as a surrogate imaging biomarker for prognosis, treatment planning and prediction of cognitive outcome in TBI. Changes to the functionally related neural networks in the resting state could also be studied during the recovery phase before and after the application of state-of-the-art neurorehabilitative interventions to assess their effectiveness. Additionally, future studies may find it useful to employ ICC maps along with DTI and brain volumetric data in order to provide a more accurate characterization of the underlying neurophysiological and structural sequalae in chronic moderate-severe TBI.

## Conclusion

In conclusion, this study demonstrated that cognitive impairments of participants with TBI associated with outcomes in cognitive measures of executive functions, verbal memory, visual memory, attention/organization, and cognitive reserve are related to altered integrity of global functional brain connectivity at rest. These findings, which are associated with differences in network connectivity in frontal and temporal cortical and subcortical networks and persist for several years after the injury, may account for part of the unaccounted variance regarding the neurophysiological substrates of cognitive deficits in chronic TBI. Larger studies are warranted to validate the above findings across the severity continuum, link ICC with anatomical connectivity patterns (e.g., using DTI) and further explore the utility of the ICC as an index of neuropathology following TBI.

## Author Contributions

NK collected neuropsychological data, collected MRI data, conducted the MRI data processing, statistical analyses, and drafted the initial manuscript. EP recruited participants, collected neuropsychological data and conducted the neuropsychological data analyses. ES advised on MRI data analyses and manuscript review. IS collected MRI data and advised on MRI data analyses, data interpretation and manuscript review. FC recruited participants, advised on data interpretation and manuscript review.

### Conflict of Interest Statement

The authors declare that the research was conducted in the absence of any commercial or financial relationships that could be construed as a potential conflict of interest.
